# Distinct Effects of Calorie Restriction and Resveratrol on Diet-Induced Obesity and Fatty Liver Formation

**DOI:** 10.1155/2011/525094

**Published:** 2011-10-01

**Authors:** Eveliina Tauriainen, Mira Luostarinen, Essi Martonen, Piet Finckenberg, Miia Kovalainen, Anne Huotari, Karl-Heinz Herzig, Anne Lecklin, Eero Mervaala

**Affiliations:** ^1^Institute of Biomedicine, Pharmacology, University of Helsinki, Biomedicum Helsinki, P.O. Box 63, 00014 Helsinki, Finland; ^2^School of Pharmacy, Pharmacology and Toxicology, University of Eastern Finland, P.O. Box 1627, 70211 Kuopio, Finland; ^3^School of Pharmacy, Pharmaceutical Technology, University of Eastern Finland, P.O. Box 1627, 70211 Kuopio, Finland; ^4^Institute of Biomedicine, Physiology, University of Oulu, P.O. Box 5000, 90014 Oulu, Finland; ^5^Biocenter Oulu, University of Oulu, P.O. Box 5000, 90014 Oulu, Finland; ^6^Department of Psychiatry, Kuopio University Hospital, P.O. Box 1777, 70211 Kuopio, Finland

## Abstract

The potential of resveratrol to mimic beneficial effects of calorie restriction (CR) was investigated. We compared the effects of both CR (70% of *ad libitum* energy intake) or resveratrol (2 g/kg or 4 g/kg food) on high-fat diet-induced obesity and fatty liver formation in C57Bl/6J mice, and we examined their effects on calorimetry, metabolic performance, and the expressions of inflammatory genes and SIRT proteins. We found that resveratrol with 4 g/kg dose partially prevented hepatic steatosis and hepatocyte ballooning and induced skeletal muscle SIRT1 and SIRT4 expression while other examined parameter were unaffected by resveratrol. In contrast, CR provided superior protection against diet-induced obesity and fatty liver formation as compared to resveratrol, and the effects were associated with increased physical activity and ameliorated adipose tissue inflammation. CR increased expressions of SIRT3 in metabolically important tissues, suggesting that the beneficial effects of CR are mediated, at least in part, via SIRT3-dependent pathways.

## 1. Introduction

Obesity, the worldwide increasing problem, associates with several metabolic abnormalities and increases the risk of chronic diseases such as type 2 diabetes, cardiovascular diseases, and certain forms of cancer [1]. Obesity is also the major risk factor for nonalcoholic fatty liver disease (NAFLD), a disease spectrum that includes hepatic steatosis, steatohepatitis, fibrosis, and liver cirrhosis [2, 3]. The fatty liver has been shown to be insulin resistant and to overproduce glucose, VLDL, CRP, and coagulation factors leading to hyperglycemia and lipid disorders [4]. 

While excessive calorie intake and subsequent obesity are associated with several health problems, calorie restriction (CR) with adequate nutrition ameliorates obesity-induced metabolic disturbances, and it has also been proven to be an effective treatment for NAFLD [5, 6]. In rodents, CR extends lifespan by up to 50% [7]. The mechanisms underlying the beneficial effects of CR is not well understood; however, accumulating evidence indicates an important role for sirtuins, a highly conserved family of NAD^+^-dependent enzymes regulating lifespan in lower organisms, as metabolic sensors and mediators of the cellular effects of CR [8].

At present, seven sirtuins (SIRT1–SIRT7) have been discovered from mammals, and of them nuclear located SIRT1 is the closest homologue of Sir2 protein that regulates the aging processes and mediates the CR-induced extension of lifespan in lower organisms [9, 10]. SIRT1 has been shown to increase cellular stress resistance and genomic stability, and it regulates cellular senescence and energy metabolism via deacetylation of the target proteins such as p53, FOXO transcription factors, and PGC-1*α* [10, 11]. It has been claimed that the beneficial cellular effects of CR are largely mediated by induction of SIRT1 whereas considerably less is known about the other members of sirtuin family. 

Three sirtuins SIRT3–SIRT5 are primarily located in mitochondrial matrix [12]. Interestingly, crosstalk has been shown between mitochondrial and nuclear sirtuins, and SIRT4 has been shown to regulate fatty acid oxidation in hepatocytes through SIRT1-dependent manner [13]. In addition, SIRT3 regulates mitochondrial function, thermogenesis, and mitochondrial fatty acid oxidation by promoting expression of mitochondrial genes [14], and by regulating the acetylation levels of metabolic enzymes, including acetyl coenzyme A synthetase 2 (AceCS2) [15, 16], and long-chain acyl coenzyme A dehydrogenase (LCAD) [17]. Moreover, SIRT3 regulates ATP synthesis by deacetylating several proteins in mitochondria electron transport Complex I [18]. SIRT3 is the only sirtuin with a reported association with the human lifespan [19, 20], making it an interesting novel target for energy homeostasis. 

As weight loss and subsequent body weight maintenance has been shown to be very difficult for obese individuals and as the compliance for low-calorie diets is often very poor in long-term clinical trials, several sirtuin-activating compounds (STACs) mimicking the beneficial effects related to CR have been developed recently [21]. Resveratrol (3,5,4-trihydroxystillbene), a natural polyphenolic compound derived from the grapes, was one of the first STACs that was shown to extend yeast lifespan through an SIRT1-dependent mechanism [21]. More recently, the beneficial effects of resveratrol were reported in mammalian cells [22]. In vivo studies with experimental obesity models have revealed that resveratrol improves health and prevents premature mortality associated with obesity [23, 24]. It is generally believed that resveratrol is mimicking the beneficial effects of CR mainly in an SIRT1-dependent manner [25]. 

The aim of the present study was to investigate the potential of long-term resveratrol supplementation to mimic the beneficial effects of CR without reducing calorie intake. We compared the effects of CR and resveratrol on diet-induced obesity and fatty liver formation. We also examined the effects of CR and resveratrol on metabolic performance, adipocyte inflammatory response, and tissue-specific protein expressions of SIRT1 and mitochondrial sirtuins SIRT3 and SIRT4. Lean mice receiving a standard low-fat diet served as healthy controls, and data from these animals were used as reference values in the present study.

## 2. Materials and Methods

### 2.1. Animals and Diets

Seven-week-old male C57Bl/6J (Charles River, Germany) mice were housed three, four, or five mice in a cage in a standard experimental laboratory, illuminated from 07.00 to 19.00, at temperature 22 ± 1°C. The protocols were approved by the Animal Experimentation Committee of the University of Helsinki (number 07–06589), Finland, and the principles of laboratory animal care (NIH publication no. 85–23, revised 1985) were followed. The mice had free access to tap water during experiment. After a one-week acclimatization period the mice (initial body weight 21.3 ± 0.1 g) were divided to five groups for 15 weeks: (1) HFD group (*n* = 18) received high-fat diet (60% of energy from fat, D12492, Research Diet Inc.; USA) *ad libitum*, (2) CR group (*n* = 18) received HFD and was kept under calorie restriction (energy intake 70% of *ad libitum* intake), (3) HFD + R2 group (*n* = 19) received HFD mixed with low-dose resveratrol (2 g/food kg; Orchid Chemicals & Pharmaceuticals Ltd., India), (4) HFD + R4 group (*n* = 19) received HFD mixed with high-dose resveratrol (4 g/kg food), (5) LFD group (*n* = 18) received low-fat diet (10% of energy from fat, D12450B, Research Diet Inc., USA) *ad libitum*. The powered high-fat diet was moistened with tap water 100 mL/kg and low-fat diet with tap water 200 mL/kg, packed in one-day portions and stored at −20°C.

### 2.2. Body Weight and Body Fat percentage Measurements

The food consumption was monitored daily and the body weight three times per week by using a standard table scale (Mettler-Toledo, Columbus, USA). The body fat percentage was analyzed from anesthetized mice by magnetic resonance imaging (MRI; Oxford Instrument, Oxford, UK) [26]. 

### 2.3. Calorimetry and Metabolic Performance

The effects of resveratrol and CR on metabolic performance, physical activity, drinking, and feeding behaviour were analysed by housing six mice from each group in a home cage-based monitoring system for laboratory animals (LabMaster TSE System, Bad Homburg, Germany). LabMaster system combines various sensors that monitor simultaneously, noninvasively, and continuously both in light and dark phases several physiological and behavioural parameters for each animal. During the experiment mice were housed in one-mouse cage after a one-week acclimatization period. The experiment was conducted during the study weeks 12–15. The measuring time was 100 hours consisting of 52 hours light phase and 48 hours dark phase. The drinking and feeding behaviour of the mice was measured by high-precision sensors attached to the top of the cage lids. The indirect calorimetry system is an open-circuit measuring system, which allowed determination of mice O_2_ consumption, CO_2_ production, respiratory exchange rate, and heat production. The infrared light-beams sensors surrounding the cage detected mice physical activity comprising ambulatory, fine, and rearing movements.

### 2.4. Tissue Sample Preparation

At the end of the calorimetric measurements the mice were decapitated. Liver, muscle (musculus quadriceps femoris), and subcutanic, epididymal, abdominal, and perirenal fat samples were dissected, washed with saline, blotted dry, and weighted. Samples were snap-frozen in liquid nitrogen and stored at −80°C until assayed.

### 2.5. Liver Histology

Formalin-fixed, paraffin-embedded liver sections (4 *μ*m) were stained with H&E. The stained sections were evaluated by a pathologist (P. Finckenberg) under a conventional light microscope in a “blinded” fashion. The samples were subjected to a semiquantitative histological analysis using the nonalcoholic steatohepatitis (NASH) Clinical Research Network scoring system for NAFLD with slight modifications for mice samples [27].

### 2.6. RNA Isolation and Gene Expression Analysis by Quantitative Real-Time Reverse Transcriptase PCR (qRT-PCR) Assay

Total RNA was isolated by TRIzol reagent (Invitrogen, Carlsbad, CA, USA). The concentration and integrity of RNA samples were analyzed by spectrophotometer (absorbance 260 and 280 nm). RNA samples were treated with DNAse I (deoxyribonuclease 1, Sigma Chemicals, St. Louis, MO) and 1 *μ*g of total RNA was reverse transcribed to cDNA by ImProm-II Reverse Transcription System (Promega, Madison, USA). The mRNA expression analysis was performed using Light-Cycler quantitative RT-PCR instrument (Roche Diagnostics, Neuilly-Sur-Seine, France). The samples were amplified with FastStart DNA Master SYBR Green 1 (Roche diagnostics) in the presence of 0.5 *μ*M of the following primers: adiponectin forward GTATCGCTCAGCGTTC and reverse GTCGTTGACGTTATCTGC; Cd68 forward CCCGAGTACAGTCTACC and reserve GTTGATTGTCGTCTGCG; leptin forward AGACCGGGCAAGAGTG and reverse GCCATAGTGCAAGGTT; MCP-1 (monocyte chemoattractant protein 1) forward CGGAACCAAATGAGATCAG and reverse TCACAGTCCGAGTCAC ; PAI-1 (plasminogen activator inhibitor 1) forward ACAGCCTTTGTCATCTCAGCC and reverse CCGAACCACAAAGAGAAAGGA; visfatin forward AGAGTGCTACTGGCTTACC and reverse CTTTCCCCCACGCTGT and 18S forward ACATCCAAGGAAGGCAGCAG and reverse TTTTCGTCACTACCTCCCCG. The quantities of the PCR products were quantified with an external standard curve amplified from purified PCR product. All the values were normalized to 18S mRNA levels.

### 2.7. Protein Isolation and Immunoblotting Analysis

Proteins from perirenal fat samples were isolated with cell extraction buffer (Invitrogen, Carlsbad, CA, USA) and complete protease inhibitors (Roche Diagnostics, Neuilly-Sur-Seine, France). Proteins from liver and muscle samples were isolated with lysis buffer (NaCl 136 mM, Na_2_HPO_4_ 8 mM, KCl 2.7 mM, KH_2_PO_4_ 1.46 mM, Tween 20 0.001% and complete protease inhibitors (Roche Diagnostics)). In immunoblotting 20 *μ*g of total protein were used and protein were separated by 8% sodium dodecyl sulphate-polyacrylamide gel electrophoresis. Proteins were transferred to a polyvinyldifluoride membrane (Immobilon-FL, Millipore, Bedford, MA, USA) and blocked for 2 h in 5% non-fat milk-TBS-0.05% Tween 20 buffer. The membranes were probed with anti-SIR2*α* (Upstate, Lake Placid, N.Y., USA) at dilution 1 : 1500, anti-SIRT3 (Abcam, Cambridge, UK) at dilution 1 : 500 and anti-SIRT4 (Abcam) at dilution 1 : 1000 in blocking buffer. Alpha-tubulin (Abcam) at dilution 1 : 2000 was used as a loading control. Horse-radish peroxidase-conjugated anti-rabbit secondary antibody (Chemicon, Temecula, CA, USA) was used at dilution 1 : 5000. The localization of horse-radish peroxidase was detected with Amersham ECL Plus (GE Healthcare, Little Chalfont, UK) according to instructions of manufacturer and visualized by FLA-9000 fluorescent image analyzer (FUJIFILM Corp., Tokyo, Japan).

### 2.8. Statistical Analysis

Data are presented as mean ± SEM. Statistically significant differences in mean values were tested by one-way ANOVA followed by the Student-Newman-Keuls test. Analyses in a function of time were done by two-way ANOVA followed by the Bonferroni test. *P* values below 0.05 were considered statistically significant. GraphPad Prism, version 4.02 (GraphPad Software, Inc., San Diego, CA, USA) was used for the statistical analyses.

## 3. Results

### 3.1. Body-Weight Gain

**Figure 1:**
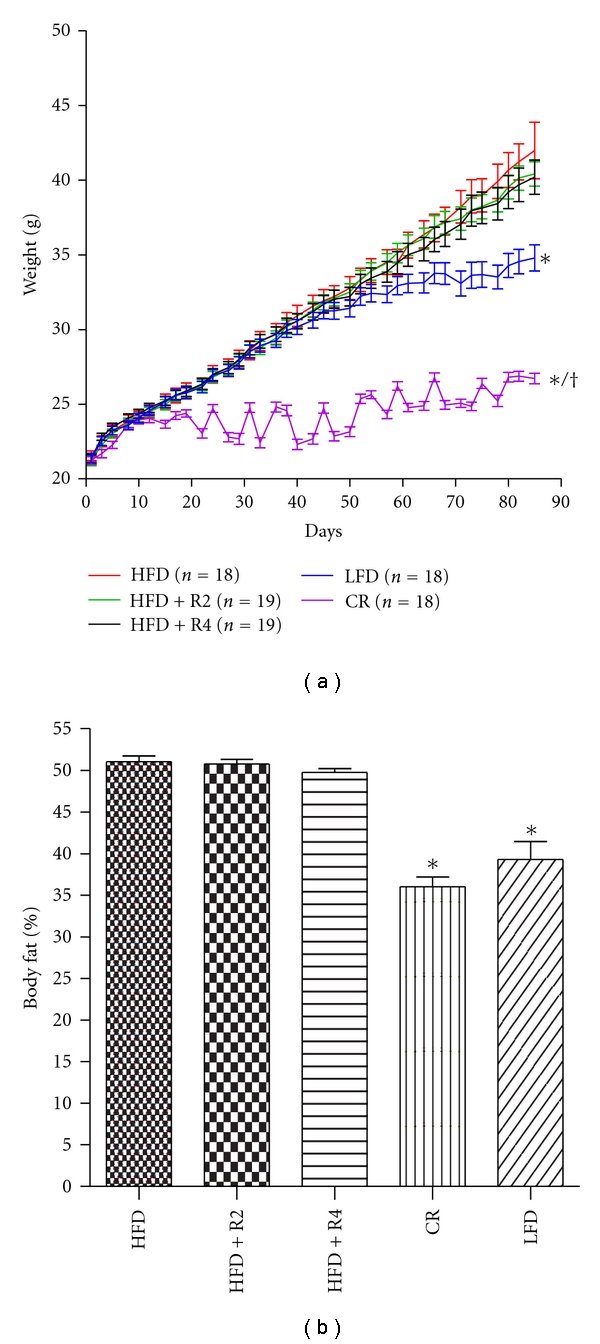
Development of body weight during 12 study weeks (a) and body fat percentage after 12 study weeks (b, *n* = 10/group). *P < 0.05 compared to HFD/HFD + R2/HFD + R4, ^†^ *P* < 0.05 compared to LFD. Data is presented as mean ± SEM.

Body weight in mice fed with HFD increased steadily during the whole follow-up period (Figure 1(a)). Neither low-dose nor high-dose resveratrol influenced body-weight gain in mice fed with HFD. The body weight in mice fed with LFD increased in parallel with HFD during the first 40 days, but thereafter remained markedly lower compared to HFD groups. There was only a modest increase in body weight in mice kept under CR, the final body weight being markedly lower compared with all other treatment groups. 

### 3.2. Body Fat Content

The body fat percentage measured by MRI was markedly higher in mice fed with HFD compared to LFD group (Figure 1(b)). Resveratrol treatments did not influence body fat percentage in mice fed with HFD. In contrast, CR decreased body fat percentage to level found in LFD-treated mice.

Neither resveratrol dosages decreased fat pad weights in mice fed with HFD whereas the weight of fat pads in LFD and CR groups were significantly lower (Table 1). The weight of different fat pads in CR group was lower compared to LFD except in abdominal fat.

### 3.3. Food, Energy, and Water Intakes

The food intake in mice fed with LFD was greater compared with other treatment groups (Table 2). However, there was no difference in energy intake between LFD and HFD groups fed *ad libitum* (Table 2). Resveratrol treatments did not influence food or energy intake. The food and energy intakes in mice under CR were approximately 70% of the *ad libitum* control values. The water intake in mice fed with LFD was higher compared with the other treatment groups (Table 2). Interestingly both low-dose resveratrol and high-dose resveratrol treatments decreased water intake whereas CR did not influence water intake. 

### 3.4. Metabolic Performance and Physical Activity

**Figure 2:**
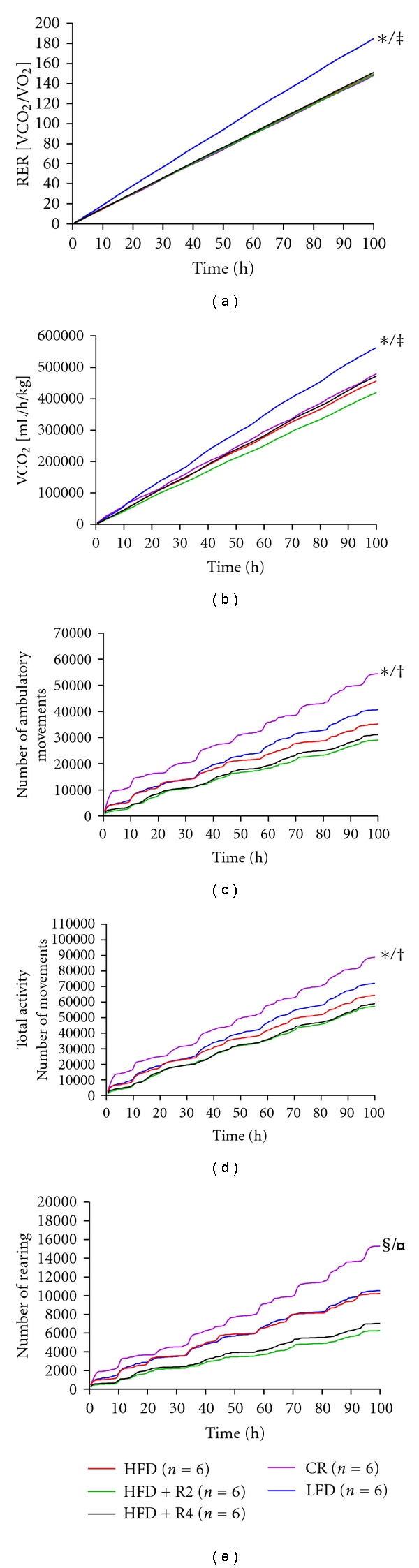
Cumulative respiratory exchange ratio (RER, a) CO_2_ (b), cumulative ambulatory (c), total activity (d), and cumulative rearing (e) of mice. *P < 0.05 compared to HFD/HFD + R2/HFD + R4, ^‡^ *P* < 0.05 CR,^†^ *P* < 0.05 compared to LFD, ^§^ *P* < 0.05 compared to HFD + R2, ^¤^ *P* < 0.05 compared to HFD + R4.

The cumulative respiratory exchange ratio (RER) and CO_2_ production in mice fed LFD was higher compared to other groups (Figures 2(a) and 2(b)). Neither resveratrol treatments nor CR influenced RER or CO_2_ production. The cumulative O_2_ and heat production did not differ between the study groups (data not shown). 

The cumulative ambulatory movements, total activity, and cumulative rearing were increased in mice kept under CR (Figures 2(c)–2(e)). The cumulative fine movements did not differ between study groups (data not shown). Resveratrol treatments tended to decrease total activity and cumulative rearing; however, the difference did not reach statistical significance (Figures 2(d) and 2(e)).

### 3.5. Liver Histology

The liver histology in mice fed with HFD showed prominent steatosis (Table 3). Furthermore, hepatocyte ballooning indicating activity of hepatocyte degeneration was found in mice fed with HFD (Table 3). Merely moderate lobular inflammation was seen in all study groups (Table 3). Complete absence of steatosis and hepatocyte ballooning was seen in the CR and the LFD groups. Resveratrol, when given at higher dosage, tended to ameliorate steatosis and hepatocyte ballooning. 

### 3.6. Adipose Tissue Inflammation and Hepatic mRNA Expressions of Visfatin and Mitochondrial Biogenesis Markers

**Figure 3:**
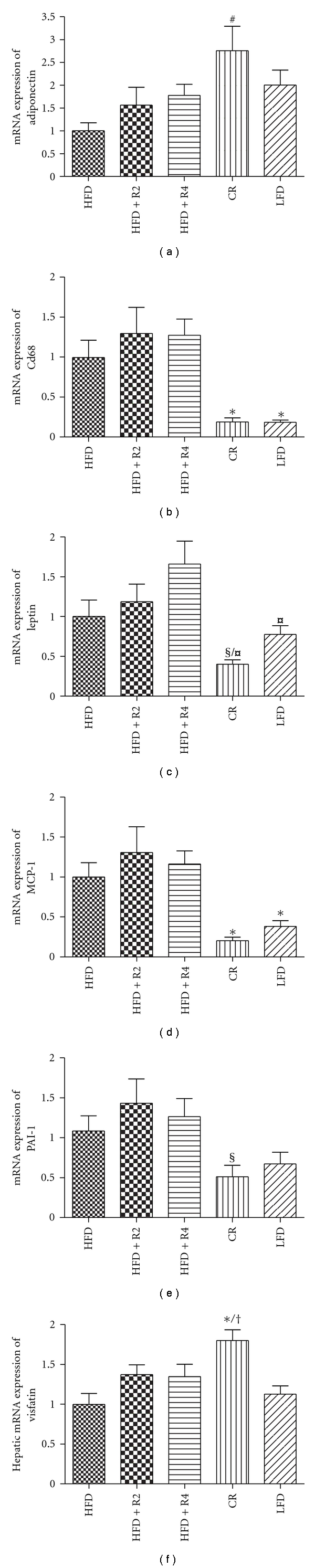
The adipose tissue mRNA expression of adiponectin (a), Cd68 (b), leptin (c), MCP-1 (d), PAI-1 (e), and hepatic mRNA expression of visfatin (d) (*n* = 12 in HFD/CR/LFD groups and *n* = 13 in HFD + R2/HFD + R4 groups). *P < 0.05 compared to HFD/HFD + R2/HFD + R4, ^#^ *P* < 0.05 compared to HFD, ^†^ *P* < 0.05 compared to LFD ^§^ *P* < 0.05 compared to HFD + R2, ^¤^ *P* < 0.05 compared to HFD + R4. Data is presented as mean ± SEM.

CR increased adipose tissue adiponectin mRNA expression and effectively decreased the mRNA expressions of inflammatory markers Cd68, leptin MCP-1, and PAI-1 in the adipose tissue (Figures 3(a)–3(e)). In addition, mice fed with LFD showed lower mRNA expression of Cd68, leptin, and MCP-1 (Figures 3(b)–3(d)). Neither low-dose resveratrol nor high-dose resveratrol influenced the mRNA expressions of adipose tissue inflammatory markers (Figures 3(a)–3(e)). There was no difference between HFD and LFD groups in the mRNA expressions of visfatin in liver (Figure 3(f)). CR markedly upregulated the visfatin mRNA expression but neither the low- or high-dose of resveratrol did not influence visfatin mRNA expression (Figure 3(f)). There were no statistical significant differences between study groups in biogenesis markers PGC-1*α*, Nrf-1, and Tfam mRNA expressions in liver (data not shown). 

### 3.7. Tissue SIRT1, SIRT3, and SIRT4 Protein Expressions

**Figure 4:**
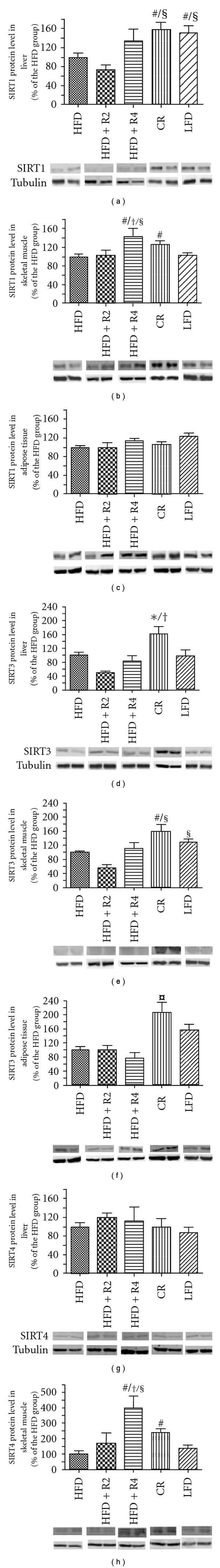
Protein expression of SIRT1 in liver (a), skeletal muscle (b), and adipose tissue (c), protein expression of SIRT3 in liver (d), skeletal muscle (e), and adipose tissue (f), and protein expression of SIRT4 in liver (g) and skeletal muscle (h) (*n* = 6/group, analyzed twice). *P < 0.05 compared to HFD/HFD + R2/HFD + R4, ^#^ *P* < 0.05 compared to HFD, ^†^ *P* < 0.05 compared to LFD, ^§^ *P* < 0.05 compared to HFD + R2, and ^¤^ *P* < 0.05 compared to HFD + R4. Data is presented as mean ± SEM.

Mice fed with HFD showed decreased SIRT1 expression in the liver as compared with LFD whereas no difference was found in skeletal muscle or adipose tissue SIRT1 expression (Figures 4(a)–4(c)). There was no difference between HFD and LFD groups in SIRT3 protein expression in liver, skeletal muscle, and adipose tissue (Figures 4(d)–4(f)). Adipose tissue SIRT4 protein expression was undetectable and no difference was seen between LFD and HFD group in SIRT4 protein expression in liver and skeletal muscle (Figures 4(g) and 4(h)).

CR increased SIRT1 expression in the liver and skeletal muscle (Figures 4(a) and 4(b)) and SIRT3 protein expression in liver, skeletal muscle, and adipose tissue (Figures 4(d)–4(f)). In addition, CR increased SIRT4 protein expression in skeletal muscle (Figure 4(h)). 

High-dose resveratrol increased SIRT1 expression in the skeletal muscle (Figures 4(b)) and tended to increase liver SIRT1 expression (Figure 4(a)). Furthermore, high-dose resveratrol increased SIRT4 protein expression in skeletal muscle and tended to increase liver SIRT1 expression (Figure 4(h)). Neither resveratrol dosages influenced SIRT3 expression (Figures 4(d)–4(f)).

## 4. Discussion

We compared the effects of calorie restriction (CR) and resveratrol supplementation on diet-induced obesity and fatty liver formation by using C57Bl/6J mice fed with high-fat diet as model of experimental obesity. The important finding of the present study was that neither low-dose nor high-dose resveratrol treatment influenced energy intake, body weight gain, body fat percentage, or metabolic performance. A modest protection against hepatic steatosis and hepatocyte ballooning was found by high-dose resveratrol treatment. In contrast, CR completely prevented development of obesity and fatty liver formation as well as adipocyte tissue inflammatory response indicating superior protection as compared to resveratrol.

Resveratrol has been widely used to mimic the physiological effects of CR [25] and to counteract the noncompliance for low-calorie diets often observed in clinical trials. Recent studies have demonstrated that CR induces SIRT1 [28, 29] a key regulator of cellular metabolism whereas resveratrol activates SIRT1 enzyme through binding to the active site of SIRT1 enzyme [21]. In the present study the resveratrol dosage and route of administration (2 or 4 g/kg food) was based on previous study by Lagouge and coworkers [24] demonstrating improvement of mitochondrial function and protection against metabolic disease in experimental obesity. Based on our calorimetric measurements and food intake recordings the above-mentioned resveratrol diet concentrations provided 135 mg/kg and 282 mg/kg daily resveratrol dosages, respectively. Surprisingly, we were unable to confirm any statistically significant reduction in body weight or adiposity in our 15-week follow-up study although a modest trend toward slightly lower body weight was noticed both in mice fed with low-dose and high-dose resveratrol. We cannot exclude the possibility that higher resveratrol dosage or longer treatment period is needed for preventing weight gain. Even though resveratrol has been shown to produce a modest decrease in body weight also in rats fed a high-fat diet [30], several studies have been unable to confirm the beneficial effects of resveratrol on body weight and obesity [23, 31]. Baur et al. [23] showed recently that low-dosage resveratrol treatment (about 20 mg/kg) increases survival of high-fat-fed mice without reducing body weight. 

Nonalcoholic fatty liver disease (NAFLD) represents a wide variety of diseases ranging from hepatocellular steatosis through steatohepatitis to fibrosis and irreversible cirrhosis [3]. Both NAFLD as well as chronic subinflammatory state caused by the release of adipokines from white adipose tissue are strongly associated with obesity, type 2 diabetes, and development of cardiovascular diseases [3, 32]. With assessment of obesity-induced fatty liver formation, we relied on previously published and validated histological scoring system [33, 34] comprising histological features of which four are evaluated semiquantitatively, namely, steatosis, lobular inflammation, hepatocellular ballooning, and fibrosis. In the present study, the steatotic change was most commonly observed in the acinar zones 2 and 3, thus resembling the adult human pattern [34]. Fibrosis and inflammation are common features in human forms of NAFLD; however, in diet-induced obese mice they were practically absent. Although the aforementioned scoring system is developed to detect NAFLD in clinical trials [33], we believe it is also suitable for screening fatty liver formation in experimental obesity. We here report that CR almost completely prevented fatty liver formation whereas resveratrol produced only a modest protection against hepatic steatosis and hepatocyte ballooning. These findings indicate superior protection against obesity-induced fatty liver formation by CR compared to resveratrol.

Chronic inflammation in the adipose tissue plays an important role in the development of obesity-related insulin resistance [35–37]. In the present study we demonstrated that CR decreased MCP-1, CD68, leptin, and PAI-1 mRNA expressions in the adipose tissue as compared to obese mice fed *ad libitum*. Furthermore, CR induced adiponectin mRNA expression in adipose tissue. These findings indicate that CR exerts anti-inflammatory effects. Neither low-dose nor high-dose resveratrol treatment influenced inflammatory response in the adipose tissue.

In agreement with previous studies [38–40], we here report that CR induced physical activity. SIRT1 is suggested to be required for increased physical activity of CR mice, as SIRT1 knock-out mice do not respond to CR with increased physical activity [41]. Consistently, we noticed marked increases in skeletal muscle and liver SIRT1 expression in mice kept under CR. Even thought the higher dose of resveratrol induced SIRT1 protein in skeletal muscle, resveratrol was unable to induce physical activity, and it tended to even decrease physical activity. This is in line with previous study by Lagouge et al. [24] demonstrating that resveratrol decreases ambulatory locomotor activity and number of rears even thought improvement in neuromuscular function was detected. Nevertheless, the reason for the lower physical activity is unclear and needs further studies. 

CR induced SIRT3 protein expression in liver, skeletal muscle, and adipose tissue suggesting that the metabolic benefits of CR are regulated also via a SIRT3-dependent manner. Both SIRT1 and SIRT3 are NAD^+^-dependent deacetylases, and the changes in NAD^+^ concentration can regulate their activity. Overexpression of visfatin (also called Nampt), which catalyze first and the rate-limiting step in NAD^+^ biosynthesis from NAM, can markedly induce SIRT1 and SIRT3 activity [42, 43]. We showed higher expression of visfatin in the CR group in liver which can also explain the higher SIRT1 and SIRT3 protein expression in liver. The inability of resveratrol to induce both visfatin and SIRT3 in metabolically important tissues can explain, at least in part, the ineffectiveness of resveratrol to mimic CR mediated health benefits. 

 Interestingly, high-dose resveratrol treatment and to a lesser extent CR increased SIRT4 protein expression in the skeletal muscle. It has been shown that SIRT4 is localized to mitochondria and mediates ADP-ribosylation of protein substrates [12]. Depletion of SIRT4 increases insulin secretion by modulation of glutamate dehydrogenase activity in pancreatic *β*-cells [44]. In addition, a recent study has provided evidence that SIRT4 depletion improves fatty acid oxidation in hepatocytes in a SIRT1-dependent manner [13]. Even thought in muscle cells SIRT4 depletion has been shown to improve mitochondrial function [13], the role of SIRT4 in skeletal muscle metabolism is still unknown and needs further studies. 

## 5. Conclusion

Using diet-induced obese mice as model of experimental obesity we demonstrated superior protection against diet-induced obesity and fatty liver formation by CR as compared to oral resveratrol supplementation. The present study also supports the notion that the beneficial metabolic effects of CR are mediated, at least in part, via SIRT3-dependent pathways. 

## Figures and Tables

**Table 1:** Fat pad weights (g).

	HFD (*n *= 12)	HFD + R2 (*n *= 13)	HFD + R4 (*n *= 13)	CR (*n* = 12)	LFD (*n* = 12)	*P* value
Subcutaneous	0.822 ± 0.073	0.752 ± 0.065	0.770 ± 0.064	0.204 ± 0.016^∗/†^	0.368 ± 0.034*	*P* < 0.001
Abdominal	0.809 ± 0.105	0.746 ± 0.083	0.732 ± 0.096	0.181 ± 0.019*	0.379 ± 0.040*	*P* < 0.001
Epididymal	2.406 ± 0.151	2.509 ± 0.124	2.463 ± 0.131	0.723 ± 0.067^∗/†^	1.348 ± 0.120*	*P* < 0.001
Perirenal	0.678 ± 0.025	0.666 ± 0.035	0.614 ± 0.040	0.182 ± 0.019^∗/†^	0.387 ± 0.034*	*P* < 0.001
Total	4.714 ± 0.262	4.673 ± 0.236	4.579 ± 0.256	1.290 ± 0.115^∗/†^	2.482 ± 0.202*	*P* < 0.001
Visceral	3.893 ± 0.215	3.921 ± 0.185	3.809 ± 0.200	1.085 ± 0.101^∗/†^	2.114 ± 0.183*	*P* < 0.001

Data is presented as mean ± SEM.

*P < 0.05 compared to HFD/HFD + R2/HFD + R4, ^†^ *P* < 0.05 compared to LFD.

**Table 2:** Food, energy, and water intakes of mice.

	HFD (*n *= 6)	HFD + R2 (*n *= 6)	HFD + R4 (*n *= 6)	CR (*n* = 6)	LFD (*n* = 6)	*P* value
Food consumption (g/day)	2.98 ± 0.20	2.90 ± 0.13	2.86 ± 010	2.16 ± 0.07*	3.86 ± 0.18^∗/‡^	*P* < 0.0001
Water consumption (mL/day)	2.16 ± 0.11	1.87 ± 0.10^‡^	1.83 ± 0.10^‡^	2.30 ± 0.07	2.61 ± 0.14*	0.0001
Energy intake (kcal/day)	14.17 ± 0.95	13.78 ± 0.63	13.55 ± 0.47	10.27 ± 0.35^∗/†^	14.85 ± 0.68	0.0004

Data is presented as mean ± SEM.

*P < 0.05 compared to HFD/HFD + R2/HFD + R4.

^†^ *P* < 0.05 compared to LFD.

^‡^ *P* < 0.05 compared to CR.

**Table 3:** Incidences of the observed histopathological lesions in liver.

	HFD	HFD + R2	HFD + R4	CR	LFD
Number of samples	10	10	10	10	10
Steatosis grade (degree/description)					
3/>66%	2	0	0	0	0
2/34–66%	5	5	3	0	0
3/5–33%	2	2	5	0	0
0/<5%	1	3	2	10	10

Lobular inflammation (degree/description)					
2/2–4 foci/20x optical field	1	0	0	0	0
1/<2 foci/20x optical field	5	8	5	4	3
0/none	4	2	5	6	7

Hepatocyte ballooning (degree/description)					
2/moderate, marked	3	0	0	0	0
1/mild, few	2	4	2	0	0
0/none	5	6	8	10	10

Fibrosis score (degree/description)					
0/none	10	10	10	10	10
